# Promoted Charge Separation and Long-Lived Charge-Separated State in Porphyrin-Viologen Dyad Nanoparticles

**DOI:** 10.1021/jacs.3c04372

**Published:** 2023-08-15

**Authors:** Bin Cai, Hongwei Song, Andjela Brnovic, Mariia V. Pavliuk, Leif Hammarström, Haining Tian

**Affiliations:** † Department of Chemistry-Ångström Laboratory, Uppsala University, Box 523, SE 751 20 Uppsala, Sweden

## Abstract

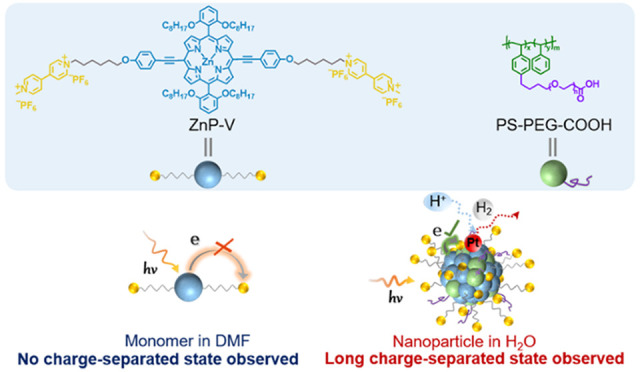

Developing light-harvesting systems with efficient photoinduced charge separation and long-lived charge-separated (CS) state is desirable but still challenging. In this study, we designed a zinc porphyrin photosensitizer covalently linked with viologen (ZnP-V) that can be prepared into nanoparticles in aqueous solution. In DMF solution, the monomeric ZnP-V dyads show no electron transfer between the ZnP and viologen units. In contrast, the ZnP-V nanoparticles in aqueous solution show fast charge separation with a CS state lifetime of up to 4.3 ms. This can be attributed to charge hopping induced by aggregation or distance modification between the donor and acceptor induced by electronic interaction. Nevertheless, the lifetime of the CS state is orders of magnitude longer than for molecular aggregates reported previously. The ZnP-V nanoparticles show enhanced photocatalytic hydrogen production as compared to the ZnP nanoparticles and still hold promise for other applications such as photovoltaic devices and photoredox catalysis.

Photoinduced long-lived charge-separated (CS) states are difficult to obtain in donor–acceptor (D–A) dyads because the subsequent charge recombination (CR) is usually quite rapid.^1^ Numerous strategies have been proposed to solve the issue. Spin control and modification of the D–A distance are two of the most adopted methods. In spin control, when the molecule is excited, a singlet CS state undergoes intersystem crossing to generate a triplet CS state in a polar solvent, and CR to the ground state is spin forbidden.^2^ The triplet CS lifetime can be further extended by application of a magnetic field.^3^ In modification of D–A distance, many studies have been focused on mimicking natural photosynthesis, in which multistep charge transfer (CT) from the light-harvesting antenna to the terminal electron acceptor with a long distance creates a long-lived CS state.^4−13^ Molecular self-assembly of dyads, triads, etc. is another method affecting CS, and this has involved holding molecules together via π–π interactions,^8,14,15^ H-bonding,^16−18^ electrostatic interactions,^19,20^ etc., in which a proper distance in the molecular assembly provides channels of charge separation and charge hopping, thus improving the CS state lifetime.^21^ In many of these cases, CS lifetimes on the order of 100 μs have been observed simply because the association constant is small and the D^+^ and A^–^ diffuse apart and bimolecular CR is limited by diffusional encounter. Very long-lived CS in molecular dyads have been reported,^22^ but other groups have questioned this conclusion and reinterpreted the data.^23,24^ Only limited work has been conducted to directly compare the photophysical properties of monomeric and aggregated states of the photosensitizer and, to our best knowledge, the CS lifetimes have never reached beyond a few microseconds in a molecular dyad light-harvesting system with visible light absorption.^15,25^

We considered that for a D–A dyad linked via a long and soft alkyl chain, aggregation could shorten the distance between D and A, therefore promoting the charge transfer between them.^26,27^ This motivated us to design a viologen-linked zinc porphyrin via a covalent hexyl chain (ZnP-V, see Figure 1) and study its photophysical properties of both the monomer state in organic solvent and an aggregated state—as nanoparticles in water. Even though no CT between porphyrin and viologen was observed by nanosecond transient absorption (ns-TA) spectroscopy when the ZnP-V monomer was measured in DMF solution, a very rapid CT from porphyrin to viologen was monitored when ZnP-V was prepared into nanoparticles in water. Moreover, a surprisingly long-lived CS state with lifetime up to 4.3 ms was observed through ns-TA spectroscopy. The phenomenon that CS states were only produced in the assembly but not in the monomeric state has been reported before. Nevertheless, CS state lifetimes achieved in similar work was only on a subnanosecond time scale.^28^ Photocatalytic hydrogen evolution experiment was also carried out in this study to show that the produced long-lived CS state in ZnP-V nanoparticles is beneficial to the photocatalytic reaction.

**Figure 1:**
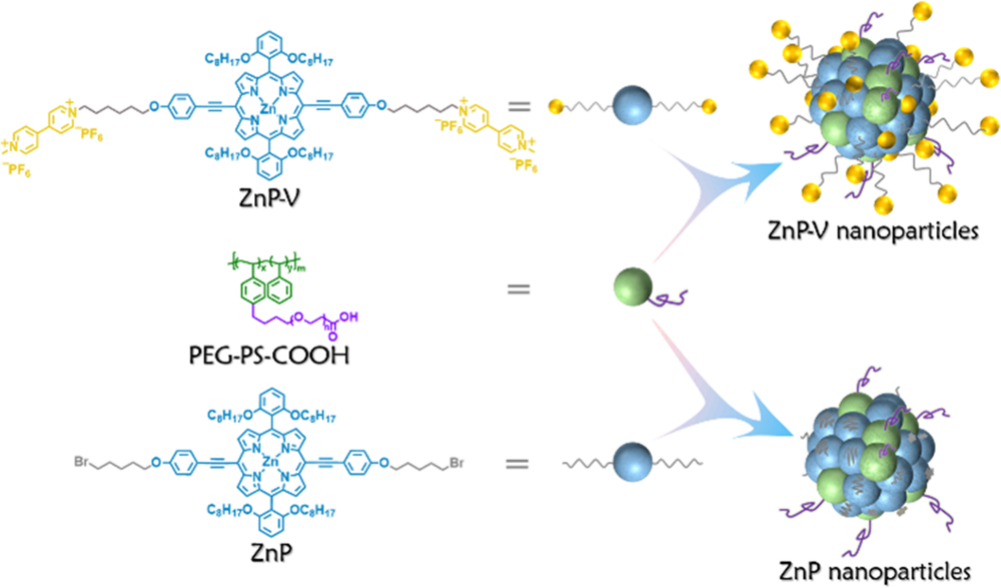
Chemical structures of ZnP-V and ZnP, and models of ZnP-V nanoparticles and ZnP nanoparticles.

Molecular structures of ZnP-V, ZnP, and schematic drawings of their corresponding nanoparticles are presented in Figure 1. Synthetic routes of ZnP-V and ZnP are provided in the Supporting Information, Scheme S1. Redox potentials of ZnP, viologen, and ZnP-V have been evaluated with cyclic voltammetry measurements and are summarized in Table S1. It was revealed that ZnP-V in DMF showed exactly the same potentials for oxidation (ZnP^+^/ZnP) and reduction (MV^2+^/MV^+^) as the individual ZnP and viologen units (Figure S1), indicating a very weak electronic coupling between the porphyrin and viologen in ZnP-V. Oxidation potential of the ZnP moiety and reduction potential of the viologen part in ZnP-V nanoparticle were provided in Figure S2 and S3. The ZnP and ZnP-V nanoparticles were prepared by a nanoprecipitation method reported before (Scheme S3).^29^ Dynamic light scattering (DLS) indicates that the prepared ZnP and ZnP-V nanoparticles give an average hydrodynamic diameter around 60 and 20 nm, respectively (Figure S4). Cryogenic Electron Microscopy (Cryo-EM) imaging of ZnP nanoparticles reveals a well-ordered polycrystalline structure with multiple lattice orientations, while ZnP-V nanoparticles tend to form a layered structure (Figure 2b and 2c). Compared to ZnP-V nanoparticles, ZnP nanoparticles in water exhibit a stronger J-aggregation with split Soret bands and red-shifted Q-bands in contrast to their DMF solutions (Figure 2a).^30−34^ The less aggregated behaviors (judging from UV–vis absorption and Cryo-EM) of ZnP-V nanoparticles indicate that the linked viologen not only points outside toward water but also mixes with ZnP inside the nanoparticles and disorganized their packings. However, ZnP-V in DMF already shows a small degree of J-aggregation as compared to ZnP in DMF from UV–vis absorption spectra, probably due to its intrinsic amphiphilic property. The linked viologen part contributes no absorption from 350 to 800 nm (Figure S5). In contrast to the photoluminescence (PL) spectra in DMF, PL spectra of ZnP and ZnP-V nanoparticles in water both are red-shifted due to aggregation with a weaker Q (0,1) PL peak caused by aggregation (Figure S6).^35^ PL excitation spectra of ZnP and ZnP-V nanoparticles monitored at different PL emission peaks are presented in Figure S7. It indicates that red-shifted PL spectra of ZnP and ZnP-V nanoparticles above around 660 nm should come from their aggregates.

**Figure 2:**
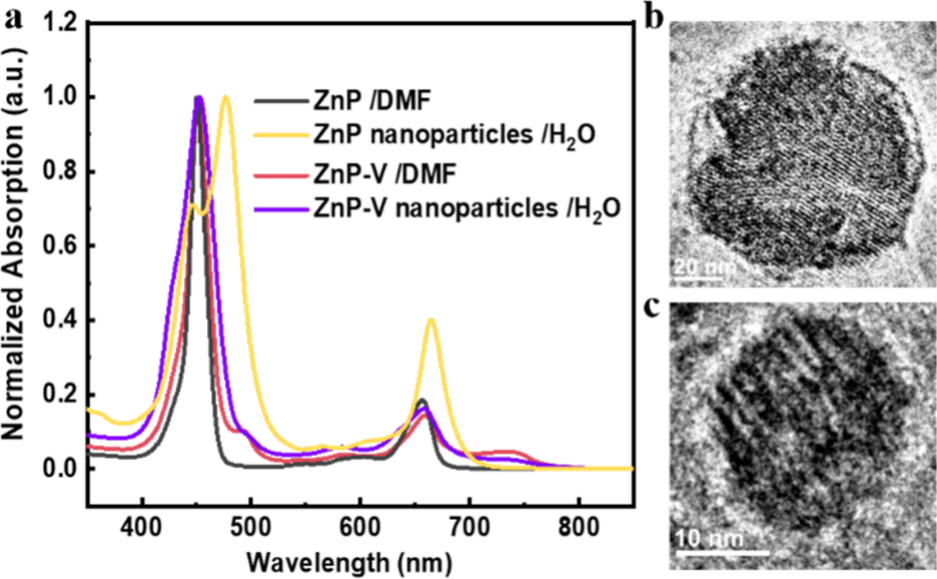
(a) Normalized UV–vis absorption spectra of the ZnP, ZnP-V, as well as ZnP nanoparticles and ZnP-V nanoparticles in H_2_O. (b, c) Cryo-EM micrographs of ZnP and ZnP-V nanoparticles, respectively.

The bimolecular reactions of photoexcited ZnP with methyl viologen (MV^2+^) and ascorbic acid, an electron donor chosen due to its suitable oxidation potential (Figure S8), in DMF were investigated by ns-TA spectroscopy. The TA spectrum at early times after excitation at 450 nm corresponds to the triplet excited ZnP (^3^*ZnP), with a broad positive band maximizing at 500 nm and the ground state bleach of the Soret and Q-bands (Figure S10). It was found that kinetic traces monitored at 500 nm exhibited negligible change after adding 0.2 M ascorbic acid (change from τ = 78.2 to 75.7 μs), while a faster decay was observed after adding 1.6 mM MV^2+^ (change from τ = 78.2 to 30.2 μs) (Figure S11). Clear signals from the reduced methyl viologen radical (MV^+•^) were observed with absorption maxima around 396 and 600 nm (Figure S10c,d),^36^ being formed on a time scale of 1 μs and decaying on the time scale of 100 μs (Figure S11b,d). Simultaneously, absorption bands of oxidized ZnP (ZnP^+^) were observed around 480 and 560 nm (Figure S10c,d, S12). When both MV^2+^ and ascorbate were present, the MV^+•^ signal was stronger and more long-lived (Figure S11c,d). These results suggest that when both MV^2+^ and ascorbate were present in the ZnP solution, the electron transfer from ^3^*ZnP to MV^2+^ should take place in the first step, followed by electron transfer from ascorbic acid to ZnP^+^. The respective Gibbs free energy reaction is summarized in Figure S9 and Table S2, S3.

After understanding the CT processes of ZnP with free viologen, we then investigated photoinduced CT between the ZnP and viologen units of ZnP-V in DMF as well as ZnP-V nanoparticles in water. The TA spectra (Figure 3a, b) and kinetic traces at 500 nm (assigned to ^3^*ZnP) (Figure 4a) exhibit no quenching after linking viologen in ZnP-V measured in DMF, suggesting that there was no direct electron transfer between the ^3^*ZnP and viologen units when both are linked by the phenylacetylene bond in the monomeric ZnP-V. Pump power dependent measurements monitored at 500 nm suggested no second-order effect was involved under these conditions (Figure S13–S14). However, the ^3^*ZnP triplet excited lifetime in ZnP-V nanoparticles (380 ns) is much shorter than that in ZnP nanoparticles (2.5 μs), indicating the effective quenching of ^3^*ZnP by viologen units in the nanoparticles (Figure 4b). Meanwhile, obvious differences in the TA spectra could be observed between ZnP and ZnP-V nanoparticles: the induced absorption band at around 400 nm of ZnP-V nanoparticles showed a blue shift compared with ZnP nanoparticles (from 415 to 395 nm). Moreover, a shoulder band at around 610 nm in ZnP-V nanoparticles formed; these two regions are the characters of reduced viologen absorption with a profile similar to Figure S11c (Figure 3c, d). No rising part of TA kinetic traces monitored at 396 nm could be observed for ZnP-V nanoparticles. This could be due to the rising part being offset by the decay of ^3^*ZnP because of the absorption spectral overlap. TA kinetic traces probed at 396 nm of the ZnP nanoparticles up to 18 μs show that a lifetime of 2.3 μs can be ascribed to the decay of ^3^*ZnP (Figure 4c). For ZnP-V nanoparticles, a biexponential fit was used and gave 380 ns (75%) + 5.8 μs (25%). The former can be ascribed to the decay of ^3^*ZnP, which is supported by the disappearance of the clear peak at 500 nm in the spectra after 1 μs (Figure 3d). The slower component can be assigned to partial charge recombination. However, ZnP-V nanoparticles exhibit a high offset at the end of the trace (Figure 4c), which should correspond to a long-lived species. Traces on a longer time scale indeed show a slower decay (Figure 4d) and a single-exponential fit to the data from 0.1 ms at 396 nm gave a lifetime of 4.3 ms.^37^ Pump power dependent measurements monitored at 396 nm shown in Figure S15 and Table S4 suggest that the component of long-lived charge-separated state is essentially the same from 0.9 to 10 mJ/pulse. The long-lived species is due to the CS state, which is seen from the simultaneous reduced viologen radical and ZnP^+^ – ZnP difference features in the ns-TA spectrum after 10 μs (Figure 3d). The additional band around 550 nm can be attributed to ZnP^+^, with possible contributions from viologen radical dimers ((V*^+^)_2_ or mixed V*^+^/V^2+^ dimer).^36,38^ Considering that ZnP^+^ absorption is obvious after 10 μs, and a similar oxidized species [ZnTPPS]^3–^ is stable for more than 0.4 s in water, we conclude that ZnP^+^ in this case is stable during the measurements.^39^

**Figure 3:**
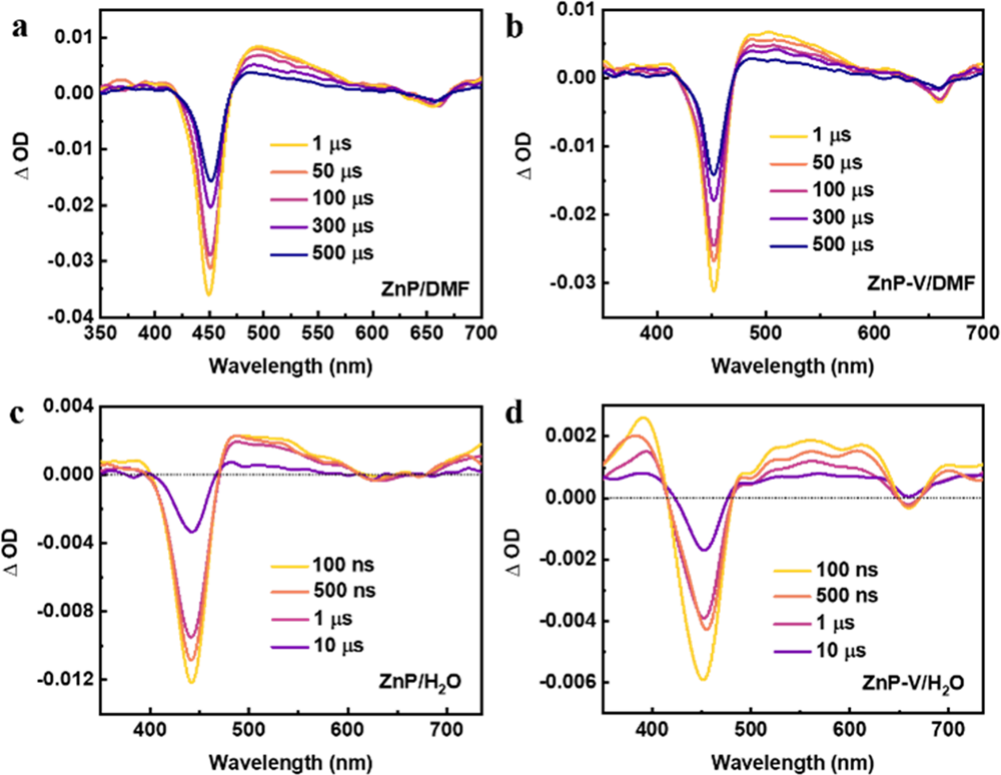
Transient absorption spectra at different delay times for (a) ZnP and (b) ZnP-V in DMF, as well as (c) ZnP and (d) ZnP-V nanoparticles in H_2_O. Excitation at 450 nm with an ∼10 ns laser pulse (pump power: 0.8 mJ/pulse).

**Figure 4:**
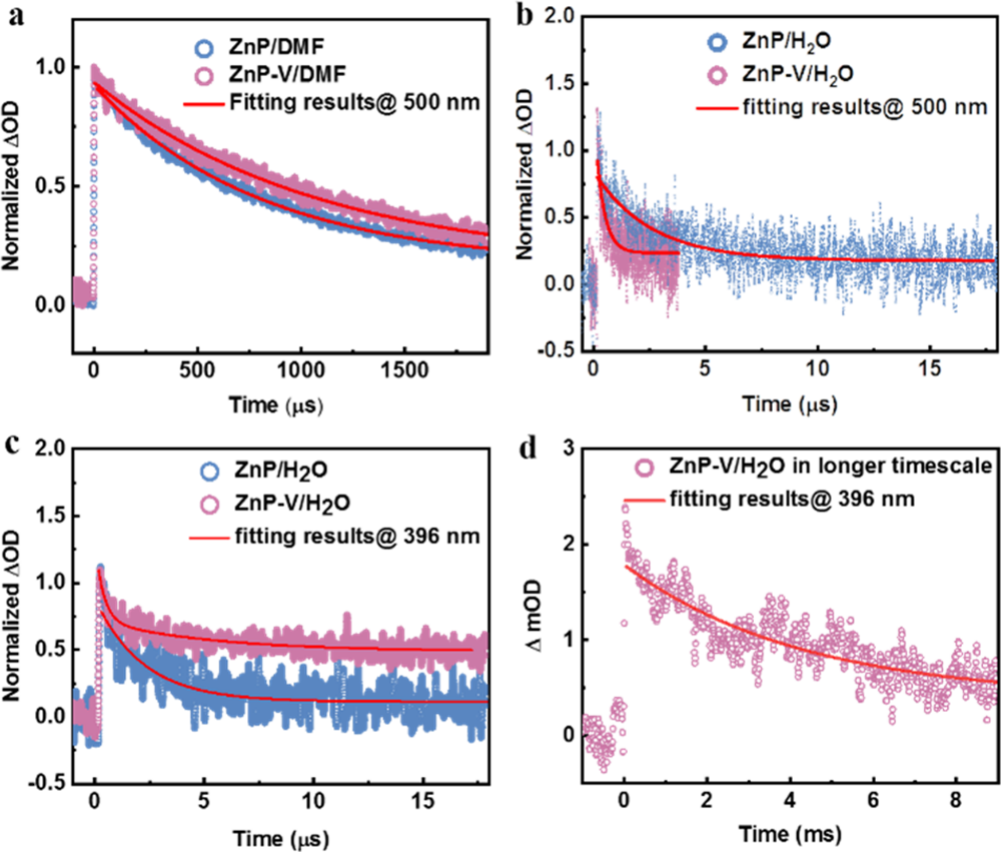
(a) Normalized TA kinetics probed at 500 nm of ZnP and ZnP-V in DMF. (b) Normalized TA kinetics probed at 500 nm of ZnP and ZnP-V nanoparticles in H_2_O; red lines are single-exponential fits with offset 0.18 (blue) and 0.23 (purple), respectively. (c) Normalized TA kinetics probed at 396 nm of the ZnP and ZnP-V nanoparticles in H_2_O; red lines are exponential fits with offset 0.11 (blue) and 0.51 (purple), respectively. (d) TA kinetic trace probed at 396 nm of the ZnP-V nanoparticles in H_2_O in longer time scale; the red line is a single-exponential fits with offset 0.4, pump power (10 mJ/pulse).

The surprisingly long-lived viologen radical may result from charge hopping inside the molecule nanoparticles because of the short intermolecular distance from molecule packing and/or the slower charge recombination due to the increased distance between the reduced viologen (V^+^) and ZnP^+^ caused by the Coulomb repulsion.^40^ Charge hopping within nanoparticles can create an electric field which could cause a Stark-effect induced spectrum. However, we did not see an obvious Stark effect spectrum from the TAS data. This can be explained by the poorly orientated electric field created within the nanoparticle (electron hopping between MV^2+^ could happen from different sides of ZnP-V molecule) and small transition dipole moment of highly symmetric porphyrin core (see DFT calculation in SI). Charge hopping between different ZnP-V nanoparticles can be excluded from previous studies due to the relatively long distance.^41,42^ Nevertheless, this generated long-lived CS state of ZnP-V nanoparticles implies a potential application of the photogenerated charges in solar energy conversion.

To verify the benefits of the obtained long-lived CS states in ZnP-V nanoparticles in solar energy conversion application, photocatalytic hydrogen evolution experiments based on ZnP-V and ZnP nanoparticles were carried out with 6 wt % Pt as the cocatalyst (cryo-EM images of the nanoparticles deposited with Pt are shown in Figure S16) and ascorbic acid as the electron donor (details in SI). Concentrations of ZnP-V and ZnP nanoparticle solution were determined to be 41 μg mL^–1^ and 58 μg mL^–1^, respectively, by the UV–vis absorption calibration curve (Figure S17). ZnP-V nanoparticles exhibited an optimal H_2_ evolution rate of 534 μmol g^–1^ h^–1^, which is 2 times higher than that of ZnP nanoparticles (Figure 5). External quantum yields based on ZnP-V and ZnP nanoparticles are 0.4% and 0.2% at 450 nm, respectively (Figure S18). In the ZnP nanoparticles, photogenerated electrons directly transferred to the Pt is the only way to proceed with the hydrogen evalution reaction. In the ZnP-V nanoparticles, photogenerated electrons might transfer to Pt directly; alternatively, the electrons transfer to the viologen unit first and then to Pt. Nevertheless, since Pt is normally located outside of the particles and there is fast electron transfer from ZnP to viologen as proved by TA measurements, the electron transfer via viologen from ZnP to Pt in ZnP-V nanoparticles should be a reasonable step.

**Figure 5:**
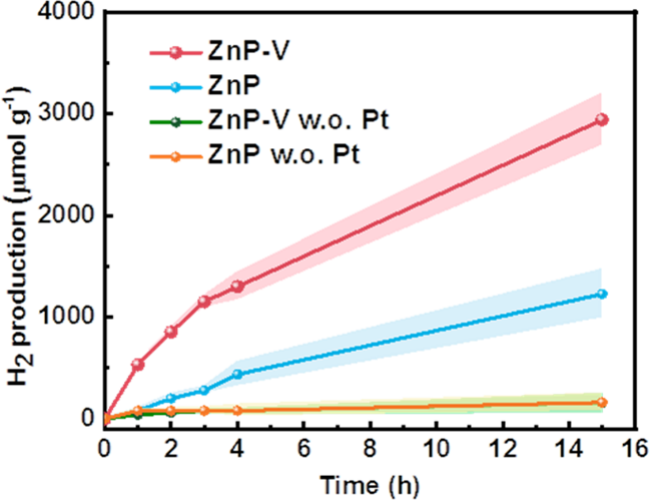
H_2_ evolution of the ZnP-V and ZnP nanoparticles with and without Pt measured under 100 mW cm^–2^ light intensity (Xe lamp, AM 1.5G filter) with 0.2 M ascorbic acid as the electron donor.

In summary, a viologen-linked zinc porphyrin via acetylene-alkyl bonds (ZnP-V) and a viologen-free zinc porphyrin (ZnP) were synthesized and prepared into nanoparticles. From TA experiments, no charge-separated product between excited ZnP and viologen in ZnP-V molecule was observed in DMF solution as evidenced by the same ^3^*ZnP lifetime between ZnP and ZnP-V in DMF, probably due to the rigid structure of the acetylene bond keeping porphyrin and viologen units far away from each other. However, a long-lived CS state lifetime up to 4.3 ms was obtained when ZnP-V was prepared into nanoparticles, which is 2 orders of magnitude longer than that of ZnP with free methyl viologen in DMF solution. Our results suggest that the nanoparticle system induced by molecule aggregation not only promotes the charge separation in the donor–acceptor dyad but also can prolong the lifetime of the CS state. Photocatalytic hydrogen production was performed to demonstrate that the long-lifetime CS state in ZnP-V nanoparticles is indeed beneficial to the photocatalytic reaction. This study emphasizes that molecule aggregation behavior controlled by nanoparticles could remarkably influence its CS state lifetime, which is of great significance in the use of molecular aggregation states as an elegant and bioinspired approach to designing other systems for solar energy conversion and storage.

## Supporting Information Available

Details of the synthesis of molecules and fabrication procedure of nanoparticles, electrochemistry and spectroelectrochemistry, TA spectroscopy, and H_2_ production procedure, et al. (PDF)
