# Supplementary material for: Promoted Charge Separation and Long-Lived Charge-Separated State in Porphyrin-Viologen Dyad Nanoparticles
Source: J Am Chem Soc. 2023 Aug 15;145(34):18687–92. doi: 10.1021/jacs.3c04372 (PMC10472426; doi:10.1021/jacs.3c04372)
Supplement: Supplementary file 1 — ja3c04372_si_001.pdf [file ja3c04372_si_001.pdf]

Promoted charge separation and long-lived charge-separated state in porphyrin-viologen dyad nanoparticles

Bin Cai [a§], Hongwei Song [a§], Andjela Brnovic [a], Mariia V. Pavliuk [a], Leif Hammarström [a*], and Haining Tian [a*]

[a] Department of Chemistry-Ångström Laboratory, Uppsala University, Box 523, SE 751 20, Uppsala, Sweden

* E-mail: leif.hammarstrom@kemi.uu.se;

* E-mail: haining.tian@kemi.uu.se.

§ B.C. and H. S. contributed equally to this paper.

1. General Information

All the chemicals were purchased from Sigma-Aldrich and used without further purification. UV-vis absorption spectra were measured on Agilent 8453 UV-visible spectroscopy. PL emission spectra and excitation spectra were measured on Spectrofluorometer FS5. Cyclic voltammetry measurements were performed on Metrohm Autolab 302N potentiostat-galvanostat controlled by NOVA software, $TBAPF_6$ as the supporting electrolyte in DMF, glassy carbon as the working electrode, platinum as the counter electrode, Ag/$AgNO_3$ as the reference electrode and ferrocene as the inner standard with a scan rate of 50 mV s^{-1}.

2. Synthesis route

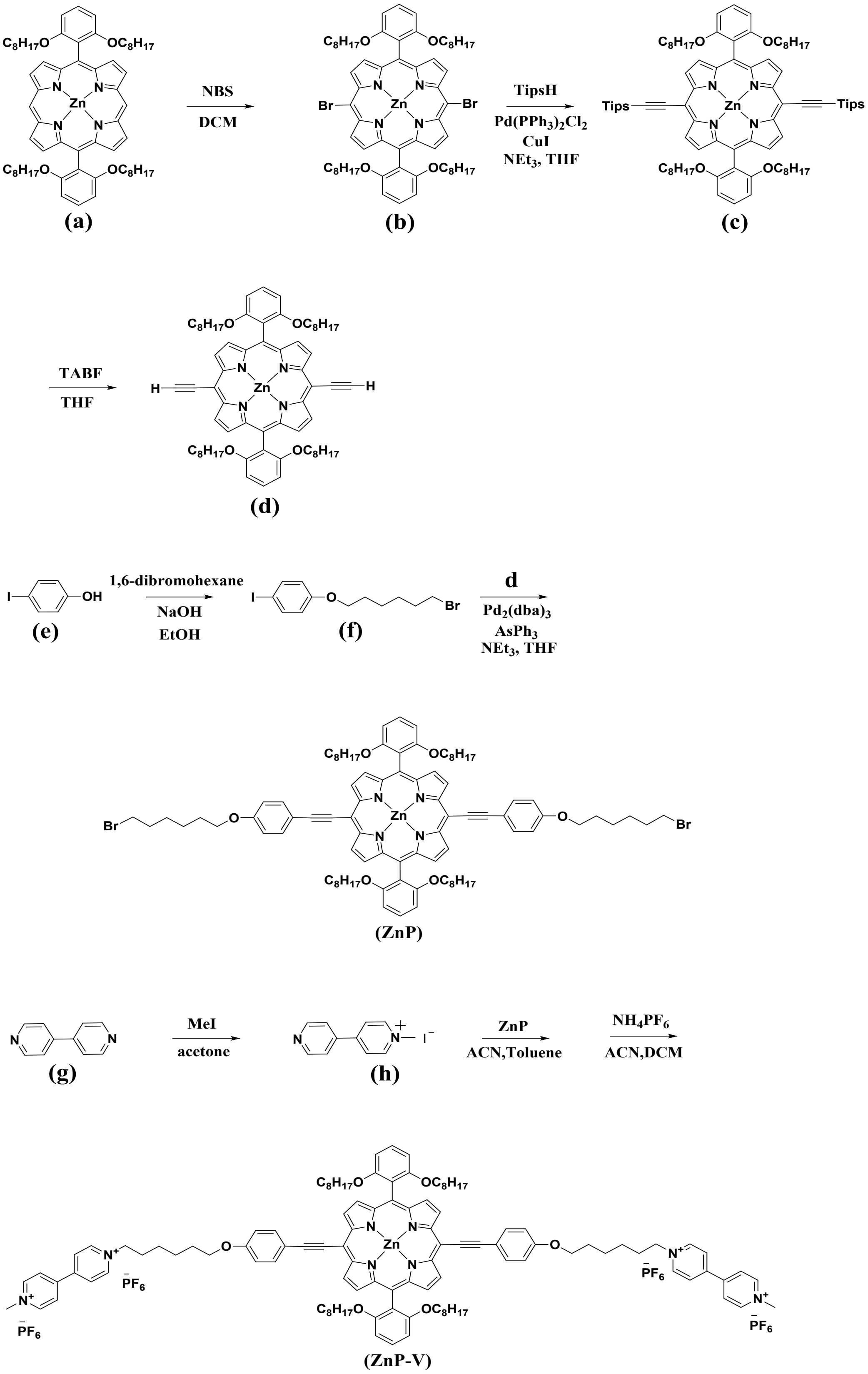

Scheme S1. Synthetic routes of the target molecule.

[5,15-Bis(2,6-dioctoxyphenyl) porphinato] Zn(II) (a)

Intermediate (a) was synthesized according to the reference[1].

[5,15-dibromo-10,20-bis(2,6-dioctoxyphenyl) porphinato] Zn(II) (b)

3 g (a) (2.9 mmol) dissolved in 150 mL DCM in an aluminum foil-covered flask. Then 1.3 g N-bromosuccinimide (2.5 eq) was added into the solution slowly under an ice-water bath and the mixture was stirred in dark overnight. The reaction was quenched with acetone, washed with water, and the organic phase was extracted with DCM. The organic solvent was removed with a rotary evaporator and purified with silica column chromatography (fluent, DCM : heptane=1:1). The product was then reprecipitated by DCM/MeOH system as a purple powder, 3 g, yield 88%. ^{1}H NMR (400 MHz, $CDCl_3$) δ 9.61 (d, J = 4.7 Hz, 4H), 8.87 (d, J = 4.7 Hz, 4H), 7.68 (t, J = 8.4 Hz, 2H), 6.98 (d, J = 8.5 Hz, 4H), 3.82 (t, J = 6.4 Hz, 8H), 0.97–0.89 (m, 8H), 0.79 (m, 8H), 0.58 (m, 8H), 0.52 – 0.44 (m, 28H), 0.35 (m, 8H). ESI-MS: m/z calculated for $C_{64}H_{80}Br_2N_4O_4Zn$=1192.4, found 1193.4 [M+1].

[5,15-bi(triisopropylsilyl)ethynyl-10,20-bis(2,6-di-octoxyphenyl)porphyrinato] zinc (II) (c)

1 g (b) (0.92 mmol) and 0.42 g triisopropylacetylene (2.5 eq), 0.19 g $Pd(PPh_3)_2Cl_2$ (0.3 eq), 0.05 g CuI (0.3 eq), 50 mL THF and 6 mL NEt_3 was gently refluxed for 4 h under nitrogen. After finishing, the mixture was washed with water and the organic solvent was removed under vacuum. The product was purified with a silica column as a green powder (fluent, DCM : heptane=1:5, v/v), 1.0 g, yield 81%. ^{1}H NMR (400 MHz, $CDCl_3$) δ 9.65 (d, J = 4.6 Hz, 4H), 8.86 (d, J = 4.5 Hz, 4H), 7.65 (t, J = 8.4 Hz, 2H), 6.97 (d, J = 8.5 Hz, 4H), 3.80 (t, J = 6.5 Hz, 8H), 1.45–1.39 (m, 2H), 0.96–0.87 (m, 8H), 0.77–0.68 (m, 8H), 0.56 – 0.29 (m, 54H). ESI-MS: m/z calculated for $C_{68}H_{124}N_4O_4Si_2Zn$=1396.8, found 1397.8 [M+1].

1-((6-bromohexyl)oxyl)-4-iodobenzene (f)

3 g 4-iodophenol dissolved in 150 mL ethanol under N_2 condition, then 5.5 g sodium hydroxide (10 eq) was added into the solution and kept stirring for 1 h. After that, 3.3 g 1,6-dibromohexane (1 eq) was added and the mixture kept refluxing for 8 hours. The crude product was washed with water and extracted with DCM, and the organic solvent was removed under a vacuum. The final product was obtained by silica column with an eluent of heptane, 3.4 g with a yield of 66%. ^{1}H NMR (400 MHz, $CDCl_3$) δ 7.56–7.49 (m, 2H), 6.68 – 6.62 (m, 2H), 3.91 (td, J = 6.4, 1.9 Hz, 2H), 3.41 (td, J = 6.8, 2.3 Hz, 2H), 1.94–1.83 (m, 2H), 1.77 (qd, J = 6.6, 2.4 Hz, 2H), 1.53–1.44 (m, 4H). EI-MS: m/z calculated for $C_{12}H_{16}BrIO$=381.9, found 382.9 [M+1].

[5,15-bis(2,6-bis(octyloxy)phenyl)-10,20-bis((4-((6-bromohexyl)oxy)phenyl)ethynyl) porphinato] Zn(II) (ZnP)

To obtain (d), 1.0 g (c) dissolved in 100 mL dry THF, 9 mL Tetra-n-butylammonium fluoride (1 M in THF, 10 eq) was added. The mixture was stirred at room temperature for 30 min under N_2. The reaction was monitored by TLC. After the reaction was completed, the reaction was quenched with water and extracted with DCM. Then, removing the organic solvent under a vacuum, only one spot was seen on TLC, and product (d) was used directly without

further purification. 0.77 g product was then transferred to an Ar replaced three-neck bottle, 0.81 g (f) (3 eq) was added and dissolved in 48 mL THF and 8 mL NEt_3. The mixture was degassed for 30 min with Ar purging. 0.26 g $Pd_2(dba)_3$ (0.4 eq) and 0.61 g $AsPh_3$ (2.8 eq) were added to the mixture. The mixture refluxed under Ar overnight. The crude product was washed with water and extracted with DCM. After removing the organic solvent under vacuum, the residue was purified by column chromatography (silica gel) with fluent of DCM: heptane=1:1 (v/v). 0.7 g dark green power was obtained with a yield of 70%. ^1H NMR (400 MHz, $CDCl_3$) δ 9.66 (d, *J* = 4.5 Hz, 4H), 8.88 (d, *J* = 4.5 Hz, 4H), 7.72 – 7.66 (m, 6H), 7.03 (d, *J* = 8.5 Hz, 4H), 6.77 (d, *J* = 8.7 Hz, 4H), 3.93 – 3.82 (m, 12H), 3.45 (t, *J* = 6.8 Hz, 4H), 1.98–1.88 (m, 4H), 1.86–1.74 (m, 4H), 1.60–1.47 (m, 8H), 1.08–0.97 (m, 8H), 0.88–0.76 (m, 8H), 0.65–0.45 (m, 44H). ESI-MS: m/z calculated for $C_{92}H_{114}Br_2N_4O_6Zn$=1592.6, found 1593.6 [M+1].

1-methyl-4,4'-bipyridine (h)

2 g 4,4'-bipyridine dissolved into 50 mL acetone, then 1.8 g iodomethane was added slowly. The mixture was kept stirring at room temperature overnight. The yellow solid powder appears after the reaction. The precipitate was obtained after filtering, washed with acetone, and dried under a vacuum. ^1H NMR (400 MHz, $CDCl_3$) δ 9.04 (d, *J* = 6.6 Hz, 2H), 8.86 – 8.81 (m, 2H), 8.51 (d, *J* = 6.3 Hz, 2H), 8.03 – 7.96 (m, 2H), 4.48 (s, 3H). EI-MS: m/z calculated for $C_{11}H_{11}IN_2$=298.0, found 171 $[M-I]^+$.

ZnP-V

500 mg ZnP and 4.7 g (h) (50 eq) were dissolved in a mixture solvent (toluene : MeOH = 1:1, v/v). The flask was covered with aluminum foil to avoid light and kept stirring for 3 days under 40 ˚C. After finishing, the solvent was removed under a vacuum. The product was washed with a large amount of toluene, then washed with a large amount of water. The crude product was then dissolved in acetonitrile, 6.1 g NH_4PF_6 (50 eq) was added, and kept stirring overnight. Removing the solvent was under vacuum, the crude product was purified with a Sephadex G-25 chromatography column, and the main band was collected. 147 mg dark green powder was obtained (yield 20%). ^1H NMR (400 MHz, d-DMSO) δ 9.53 – 9.50 (m, 4H), 9.41 (d, *J* = 6.3 Hz, 4H), 9.28 (d, *J* = 5.9 Hz, 4H), 8.80 (d, *J* = 6.0 Hz, 4H), 8.76 (d, *J* = 6.3 Hz, 4H), 8.65 (d, *J* = 4.4 Hz, 4H), 7.99 – 7.91 (m, 6H), 7.74 (t, *J* = 7.8 Hz, 4H), 7.14 (dd, *J* = 17.9, 8.4 Hz, 4H), 4.42 (s, 6H), 3.87 (s, 12H), 3.45 – 3.39 (m, 4H), 2.10 – 2.02 (m, 4H), 1.86 – 1.80 (m, 4H), 1.50 – 1.41 (m, 8H), 0.99 – 0.91 (m, 8H), 0.69 – 0.64 (m, 8H), 0.59 – 0.54 (m, 44H). ^{13}C NMR (400 MHz, d-DMSO) δ 159.731, δ 151.175, δ 159.133, δ 149.122, δ 147.179, δ 146.363, δ 133.350, δ 132.086, δ 131.962, δ 127.090, δ 126.614, δ 120.683, δ 115.568, δ 105.766, δ 68.087, δ 61.794, δ 48.261, δ 40.705, δ 40.657, δ 40.495, δ 40.286, δ 40.074, δ 39.403, δ 33.061, δ 31.572, δ31.471, δ31.259, δ28.876, δ28.831, δ28.706, δ25.311, δ22.483, δ22.359, δ14.368, δ14.284, δ14.269. ESI-MS: m/z calculated for $C_{114}H_{136}F_{24}N_8O_6P_4Zn$, $[(M-4PF_6)/4]^+$= 444.27, found 444.27.

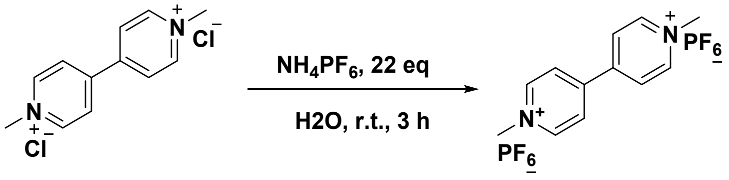

Scheme S2. Synthetic route of 1,1'-dimethyl-[4,4'-bipyridine]-1,1'-diium hexafluorophosphate (MV used in this study)

1,1'-dimethyl-[4,4'-bipyridine]-1,1'-diium hexafluorophosphate was synthesized according to reference, and obtained as the white powder.[2]

3. Molecule nanoparticles preparation method

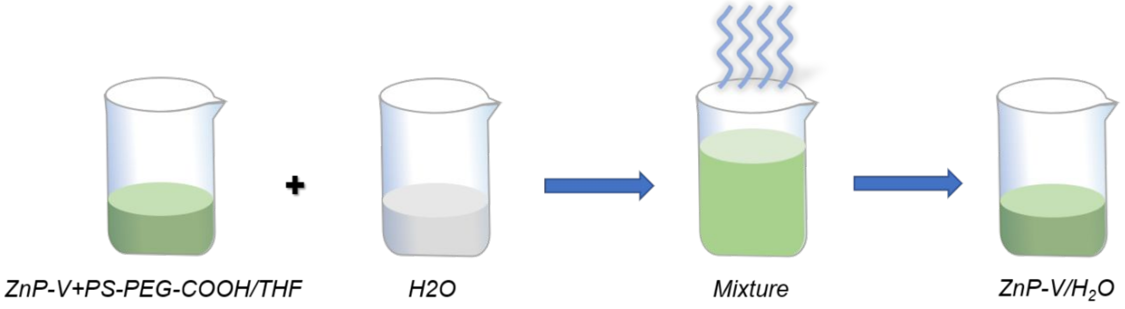

Scheme S3. Scheme of preparation procedure of the molecule nanoparticles

First, 4 mg mL^{-1} ZnP-V in acetonitrile and 1 mg mL^{-1} PS-PEG-COOH in THF are prepared as the stock solution, respectively. Dilute the as-prepared stock solution with THF to be 50 µg mL^{-1} ZnP-MV and 50 µg mL^{-1} PS-PEG-COOH mixture. Sonicating the organic solution for 5 minutes to fully mix the surfactant. Then, the prepared organic solution was poured into water (1:1, v:v) rapidly with sonification. The obtained mixture was put in the fume hood under dark conditions, and the THF evaporated slowly. Finally, water-dispersed molecule nanoparticles were obtained after filtering. ZnP nanoparticles were prepared in the same way by preparing the ZnP stock solution in THF.

4. Molecule nanoparticle concentration calibration

Taking 200 µL water dispersed ZnP-V or ZnP molecule nanoparticle into a 4 mL vial, and evaporating the water under vacuum. Dissolving the dry solute with 4 mL ACN or THF, and measuring the UV-vis absorption spectra. Comparing the obtained absorbance at 455 nm with the standard calibration curve.

5. Hydrogen evolution procedure

1.5 mL ZnP-V nanoparticles/H_2O and 6wt% Pt (mass ratio to the nanoparticle, in the form of H_2PtCl_6) was added into a 9 mL vial. The mixture was then purged with Ar for 30 minutes. 0.5 mL 2M ascorbic acid (pH = 4.3, tuned by 2M NaOH) was added after being purged with Ar for 30 minutes. The sealed vial was illuminated with a Xenon light (CEL-HXF300, the light intensity calibrated to be 1 sun intensity, 100 mW·cm^{-2}). The produced hydrogen was detected with the GC (Thermo Scientific, Trance 1300) by injecting 100 µL headspace gas, and the amount was calculated by a calibration plot.

6. Nanosecond Transient Absorption Measurements

For nanosecond transient absorption measurements, optical excitation was performed by using the third harmonic output of a frequency-doubled Q-switched Nd: YAG laser combined with an OPO to generate 450 nm excitation pulses. For time-resolved spectra and kinetic traces on nano-to-microsecond time scales, Eskpla NT340 was used to give 450 nm. The laser was coupled to an LP 920 detection system (Edinburgh Instruments) equipped with a pulsed XBO 450 W xenon Arc Lamp (Osram), which can provide white light for probing. An iStar CCD camera (Andor Technology) and an LP920-K photomultiplier (PMT) detector connected to a

Tektronix TDS 3052 500 MHz 5 GS/s oscilloscope were used for transient signal detection. Transient absorption data were acquired using LP 900 software and processed using Origin 2018 software. For kinetic traces on milli-second time scales, a Quantel, Brilliant B laser with Opotek OPO was used to provide 450 nm, 0.8, 2.4, 5.6, 10 mJ pulses. The probe light was single wavelength and provided using an un-pulsed 150 W Xe lamp in a flash photolysis spectrometer (Applied Photophysics LKS.60). Two monochromators were used to minimize sample excitation by probe light: the first monochromator was set to the desired detection wavelength before reaching the sample, and the second monochromator was placed after samples. The absorption difference of samples at specified wavelengths can be monitored with a PMT Hamamatsu R928 detector and digitized using an Agilent Technologies Infinium digital oscilloscope (600 MHZ). Transient absorption data were acquired within the Applied Photophysics LKS software package. All transient absorption and emission measurements were carried out at room temperature, and a 1.0 cm path length quartz cell cuvette was used for the measurements, and before measurements, all solutions were degassed with Ar.

7. Cryogenic Electron Microscopy (Cryo-EM) Measurements

Cryo-EM was performed on a Zeiss Libra 120 transmission electron microscope (Carl Zeiss AG, Oberkochen, Germany). The microscope operated at 80 kV and in zero-loss bright-field mode. Low-dose conditions with a BioVision Pro-SM Slow Scan CCD camera (Proscan Elektronische Systeme GmbH, Scheuring, Germany) were used to obtain digital images. R1.3/1.2 300 mesh grids (QuantiFoil) were glow-discharged (20 mA for 120 s) on a PELCO EasiGlow. Concentrations of samples were as following: ZnP NPs (418 μg/mL), ZnP-V NPs (225 μg/mL). Each mixture (3 μL) was applied onto grids before plunge-freezing into liquid ethane in a Vitroblot Mark IV robot (FEI/Thermo Fisher Scientific) operating at 25 °C, 95% humidity (blot time of 4 s). Samples were vitrified in liquid ethane and transferred to the microscope, continuously kept below –160 °C and protected against atmospheric conditions.

8. Supplementary Figures

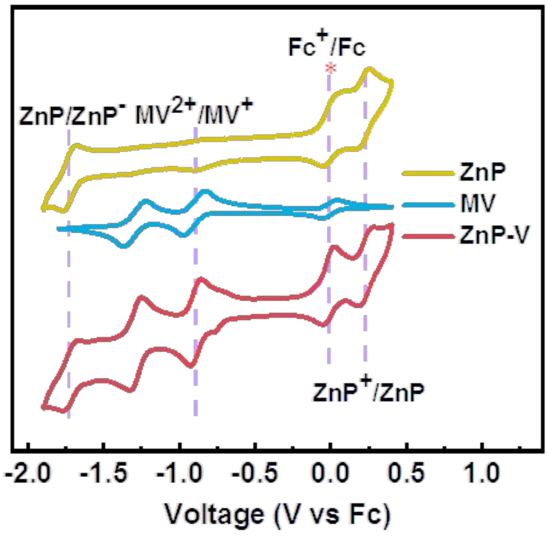

Figure S1. Cyclic voltammetry of ZnP, methyl viologen, and ZnP-V in DMF with 0.1 M $TBAPF_6$ as supporting electrolyte and with $Ag/AgNO_3$ as the reference electrode (ferrocene was used as the inner standard, the same redox peak of the ferrocene obtained, indicating that the stable of the reference electrode during the measurement), DMF as the solvent, scan rate is 50 mV s^{-1}.

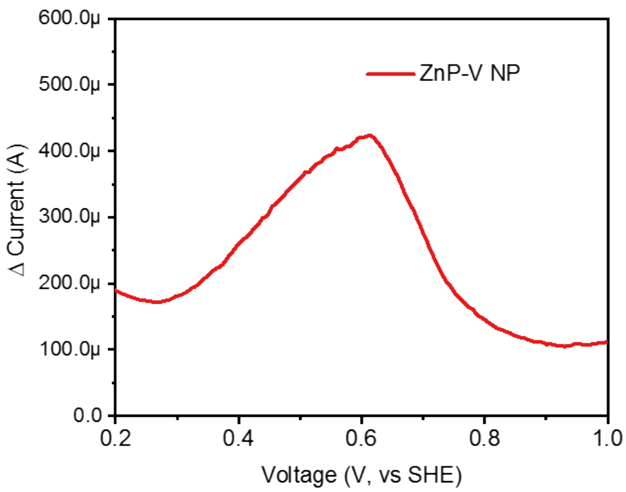

Figure S2. Differential pulse voltammetry of the ZnP-V NP drop-dried on carbon cloth as working electrode, Pt wire as the counter electrode, Ag/AgCl (3M KCl) as the reference electrode, 0.2 M KCl as the supporting electrolyte, scan rate 5 mV s^{-1}, oxidation from 0.2V to 1V.

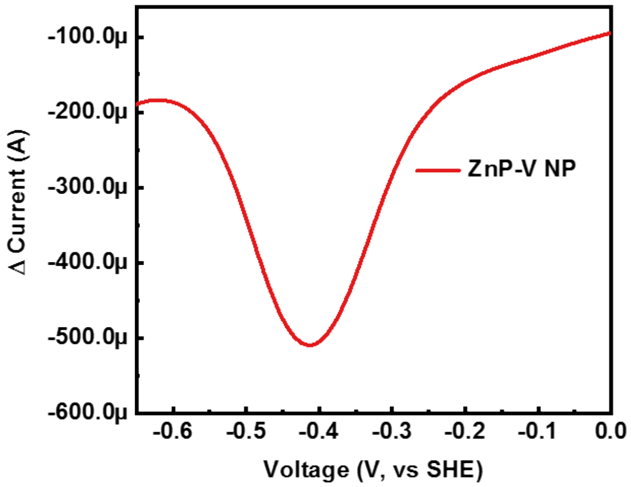

Figure S3. Differential pulse voltammetry of the ZnP-V NP drop-dried on carbon cloth as working electrode, Pt wire as the counter electrode, Ag/AgCl (3M KCl) as the reference electrode, 0.2 M KCl as the supporting electrolyte, scan rate 5 mV s^{-1}, reduction from 0 V to -0.7 V.

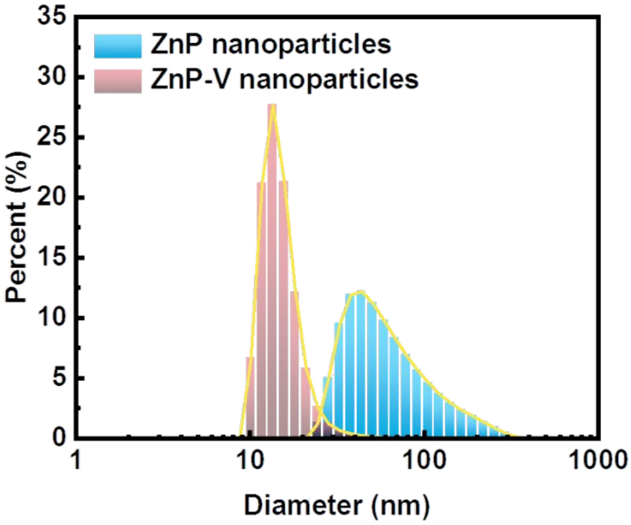

Figure S4. DLS average hydrodynamic diameter of the as-prepared ZnP and ZnP-V nanoparticles in H_2O.

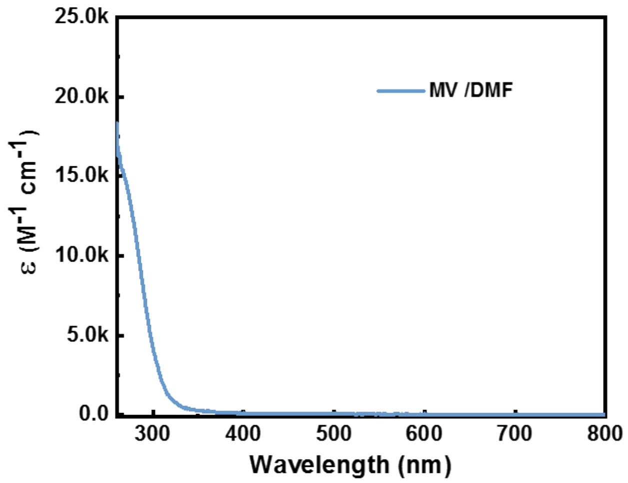

Figure S5. The absorption spectrum of synthesized methyl viologen measured in DMF.

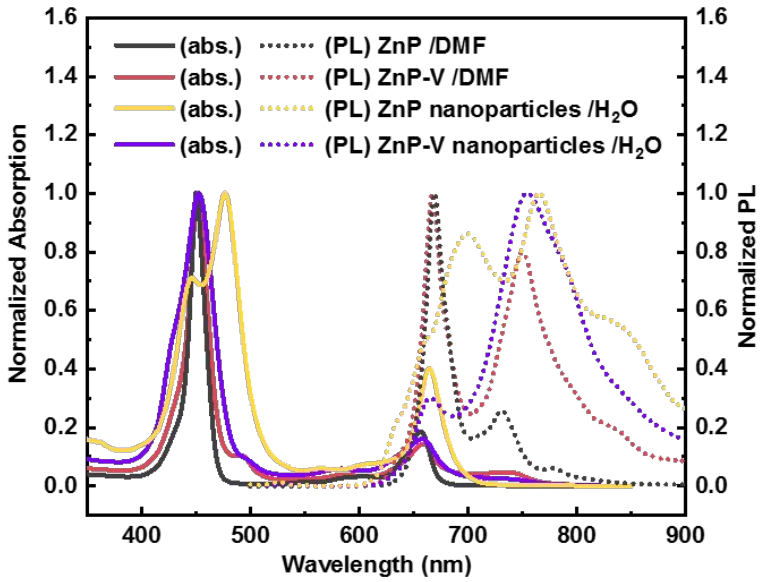

Figure S6. Normalized UV-vis absorption (solid line) and PL emission spectra (dash line) of the ZnP and ZnP-V in DMF, as well as ZnP nanoparticles and ZnP-V nanoparticles in H_2O.

.

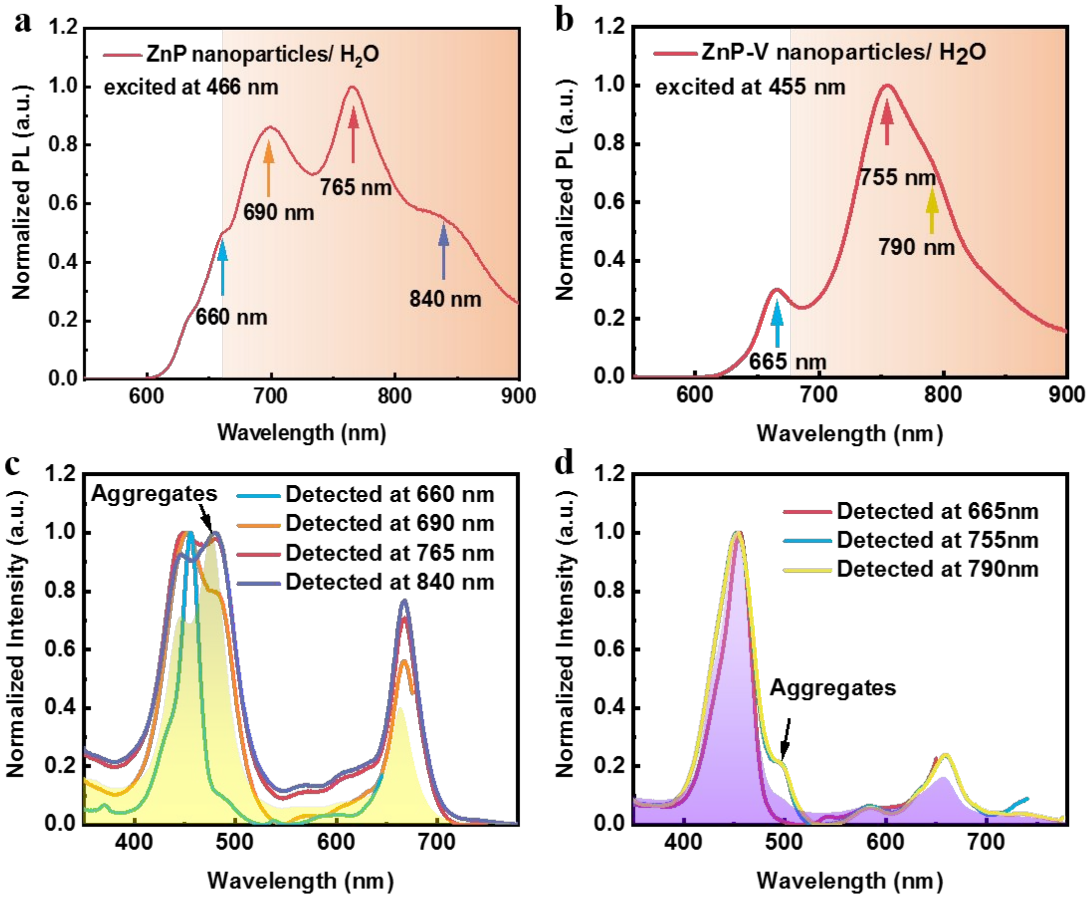

Figure S7. (a) The PL emission spectrum of the ZnP nanoparticles measured in H_2O, excited at 466 nm, the arrows indicate the detected wavelengths of the PL excitation spectrum; (b) PL emission spectrum of the ZnP-V nanoparticles measured in H_2O, excited at 455 nm, the arrows indicate the detected wavelengths of the PL excitation spectrum; (c) Normalized PL excitation spectra of ZnP nanoparticles in H_2O detected at four different PL emission peaks (corresponding to Fig. S5a), the yellow area is the corresponding absorption spectrum of ZnP nanoparticles in H_2O; (d) Normalized PL excitation spectra of ZnP-V nanoparticles in H_2O detected its three different PL emission peaks (corresponding to Fig. S5b), the purple area is the corresponding absorption spectrum of ZnP-V nanoparticles in H_2O.

PL excitation spectra of ZnP nanoparticles and ZnP-V nanoparticles monitored at different PL emission peaks were presented in Figure S4. When detected at 660 nm, PL excitation spectrum of ZnP nanoparticles is closer to UV-vis absorption spectrum of ZnP measured in DMF. As the detected wavelength redshifted, PL excitation spectrum of ZnP nanoparticles is closer to UV-vis absorption spectrum of ZnP nanoparticles measured in H_2O. Similarly, when detected at 665 nm, PL excitation spectrum of ZnP-V nanoparticles is also closer to UV-vis absorption spectrum of ZnP measured in DMF. As the detected wavelength redshifted, PL excitation spectrum of ZnP-V nanoparticles is then closer to UV-vis absorption spectrum of ZnP-V nanoparticles measured in H_2O. These results indicated that redshifted PL spectra of ZnP and ZnP-V nanoparticles above around 660 nm should come from their aggregates.

Table S1. Redox potentials of ZnP, viologen and ZnP-V vs. SHE.

$E_{MV^{2+}/MV^{+}}$	$E_{ZnP^{+}/ZnP}$	$E_{ZnP/ZnP^{-}}$	$E_{ZnP^{+}-V/ZnP-V}$	$E_{ZnP-V/ZnP^{-}-V}$	$E_{ZnP^{+}-V/ZnP^{*}-V}$
-0.42 V	0.69 V	-1.26 V	0.69 V	-1.26 V	-0.85 V

All redox potentials are relative to the SHE, with a formula as follows:

E (vs SHE) = E (vs Ag/AgNO$_3$) – E (Fc vs Ag/AgNO$_3$) + E (Fc vs SHE)

Here, in the right equation, the first part is obtained from the measured CV value in DMF, the second part is obtained from directly adding ferrocene into the solution as the inner standard when measuring the sample in DMF, the third part E(Fc vs SHE) = 0.47 V when measured in DMF, which obtained from the reference.[3]

To calculate the reduction potentials of the photoinduced ET reaction, the following equation was used:

$$E_{ZnP^{+}-V/ZnP^{*}-V} = E_{ZnP^{+}-V/ZnP-V} - E_{0-0,triplet}$$
$$= E_{ZnP^{+}-V/ZnP-V} - E_{0-0,singlet} + \Delta E_{ST}$$

where $E_{0-0,triplet}$ and $E_{0-0,singlet}$ are the 0-0 energies of the triplet state and singlet state of the ZnP respectively: $E_{0-0,singlet}$ was deduced from the wavelength at the intersection of the normalized UV-vis absorption spectrum and PL emission spectrum. It has been reported that the energy gap (ΔE_{ST}) between S$_1$ and T$_1$ states of the zinc porphyrin is around 0.33 eV,[4-6] and we assumed the same value for ZnP-V.

Gibbs free energy ΔG_1 for oxidation of the excited triplet state ZnP* by V^{2+} is calculated from:

$$\Delta G_1 = -(E_{MV^{2+}/MV^{+}} - E_{ZnP^{+}-V/ZnP^{*}-V})F$$

Gibbs free energy ΔG_2 for reduction of the ZnP^{+}-V by ascorbic acid is calculated from:

$$\Delta G_2 = -(E_{ZnP^{+}-V/ZnP-V} - E_{Asc^{+}/Asc})F$$

When considering the Coulombic repulsion interaction between oxidized ZnP^{+} and reduced viologen MV^{+}, as well as the solvent influence, Gibbs energy of photoinduced electron-transfer process between ZnP and viologen was calculated as below[7, 8]:

$$\Delta G_{ET} = e(E_{ZnP^{+}/ZnP} - E_{MV^{2+}/MV^{+}}) - E_{0-0} + \frac{e^2}{4\pi r_{ZnP-V}\varepsilon_0\varepsilon_S} + \frac{e^2}{8\pi\varepsilon_0}\left(\frac{1}{r_{ZnP}} + \frac{1}{r_{MV}}\right)\left(\frac{1}{\varepsilon_S} - \frac{1}{\varepsilon_P}\right)$$

$$C = \frac{e^2}{4\pi r_{ZnP-V}\varepsilon_0\varepsilon_S}$$

$$S = \frac{e^2}{8\pi\varepsilon_0}\left(\frac{1}{r_{ZnP}} + \frac{1}{r_{MV}}\right)\left(\frac{1}{\varepsilon_S} - \frac{1}{\varepsilon_P}\right)$$

$$\Delta G_{ET} = e(E_{Ox} - E_{\mathrm{Re}d}) - E_{0-0} + C + S$$

Wherein, ΔG_{ET} is the Gibbs energy of photoinduced electron-transfer process between ZnP and viologen; r_{ZnP-V} is the distance from the ZnP center to viologen center (23.5 Å),

estimated from the Chem 3D; $\varepsilon_0 \approx 8.854\times10^{-12} C^2 J^{-1} m^{-1}$ is the vacuum permittivity; $\varepsilon_S \approx 78.4$ is the relative permittivity of water; e is the elementary charge; $\varepsilon_P \approx 38.4$ is the relative permittivity of DMF; r_{ZnP} and r_{MV} is the ionic radii of ZnP (13.5 Å) and MV (6.7 Å), estimated from Chem 3D.

For both ZnP-V/ DMF and ZnP-V NP/ H_2O, the electrochemical data were recorded in the same electrolyte as are used in the photoelectrochemical and transient studies, therefore there are no difference in solvation energy between the photogenerated charge separated state and the electrochemically oxidized and reduced species (S = P), making the solvation term (*S*) in equation zero.

Table S2 Calculated Gibbs energy of photoinduced electron transfer from the ZnP to viologen in water

	$E_{ZnP^+/ZnP}$	E_{MV^{2+}/MV^+}	$E_{0-0,triplet}$	C	S	ΔG_{ET}
ZnP-V	0.62 V	-0.41 V	1.54 eV	0.01 eV	0 eV	-0.5 eV

While driving force of the photoinduced electron transfer from the ZnP to viologen in DMF is summarized as follows.

Table S3 Calculated Gibbs energy of photoinduced electron transfer from the ZnP to viologen in DMF

	$E_{ZnP^+/ZnP}$	E_{MV^{2+}/MV^+}	$E_{0-0,triplet}$	C	S	ΔG_{ET}
ZnP-V	0.69 V	-0.42 V	1.54 eV	0.02 eV	0 eV	-0.41 eV

In ZnP-V nanoparticles, the slightly increased driving force (90 mV) mainly come from a more negative oxidation potential of ZnP moiety, which is due to different electrolytes or/and solvents used for these experiments, or the molecule aggregation.

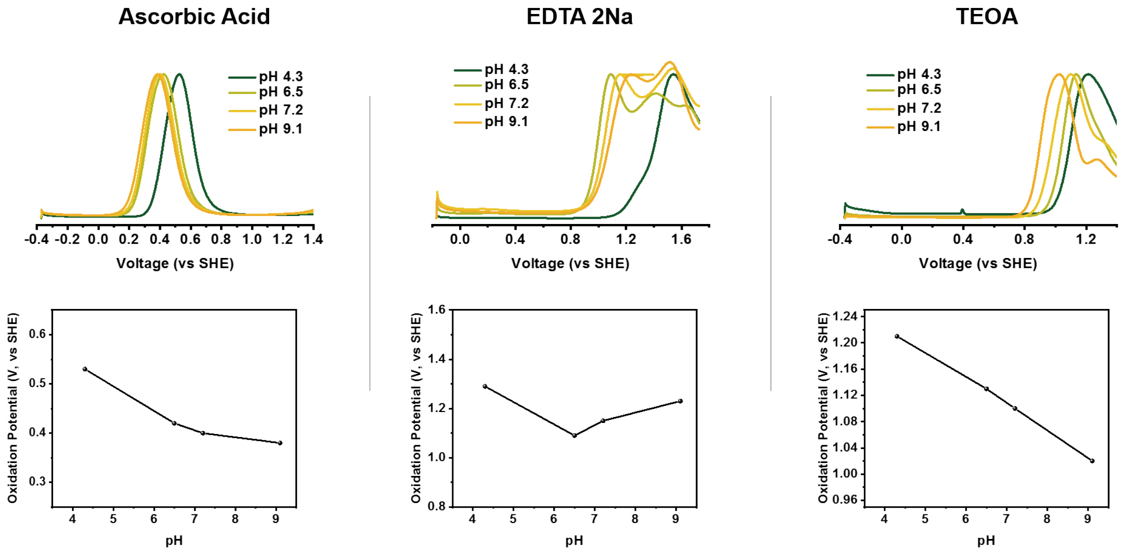

Figure S8. Differential pulse voltammetry of different electron donors was measured under different pH, different pH varied by phosphate buffer, all measurements were performed in water with 0.1 M KCl as the supporting electrolyte, Ag/AgCl (3M KCl) as the reference electrode, a scan step of 4 mV, modulation amplitude of 50 mV, modulation time of 50 ms, internal time of 500 ms and a scan direction from negative to positive to detect the oxidation processes (the first oxidation peak is extracted as the first oxidation potential).

To complete the photocatalytic hydrogen production, a sacrificial donor is usually required. Oxidation potentials of several different sacrificial donors under various pH values were evaluated, and ascorbic acid was chosen due to its suitable oxidation potential of 0.31 V vs. SHE at pH 4.3.

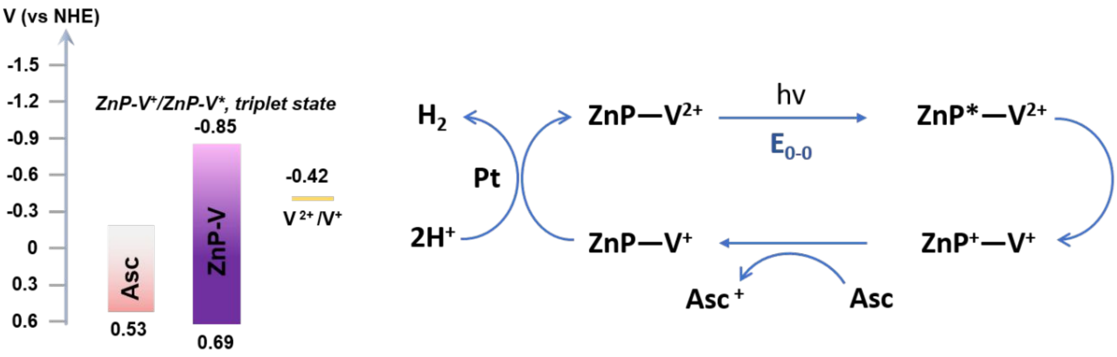

1. $ZnP \longrightarrow ZnP^*$

2. $ZnP^* + V^{2+} \longrightarrow ZnP^+ + V^+$

3. $ZnP^+ + Asc \longrightarrow ZnP + Asc^+$ $\quad \Delta G_1 = -(E_{ZnP^+/ZnP} - E_{ASC^+/ASC})F = -15.4kJ$

4. $2V^+ + 2H^+ \xrightarrow{Pt} V^{2+} + H_2$ $\quad \Delta G_2 = -2 * (E_{H^+/H_2} - E_{V^{2+}/V^+})F = -81.1kJ$

Figure S9. Energy level alignment diagram and working principle of the as-prepared photocatalysis system.

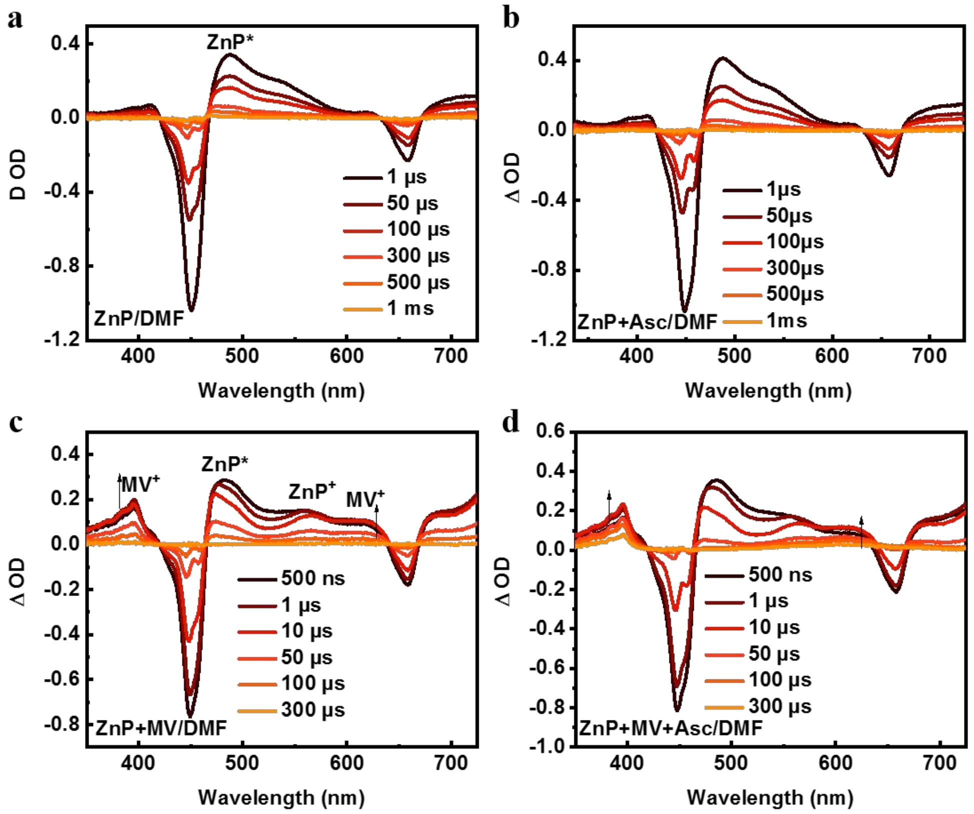

Figure S10. Transient absorption spectroscopy of (a) ZnP, (b) ZnP with 0.2 M ascorbic acid, (c) ZnP with 1.6 mM methyl viologen, and (d) ZnP with both 1.6 mM methyl viologen and 0.2M ascorbic acid at different delay times after excitation with a ~10 ns laser pulse at 450 nm, pump power: 10 mJ/pulse.

Note: The main purpose of this part is to **qualitatively** investigate after ZnP being excited, which of the following step would happen first: reduction quenching by viologen or oxidation quenching by ascorbic acid. So, ZnP/DMF solution was prepared with that a concentration of absorbance at 450 nm is around 1.8. The same for Figure S11.

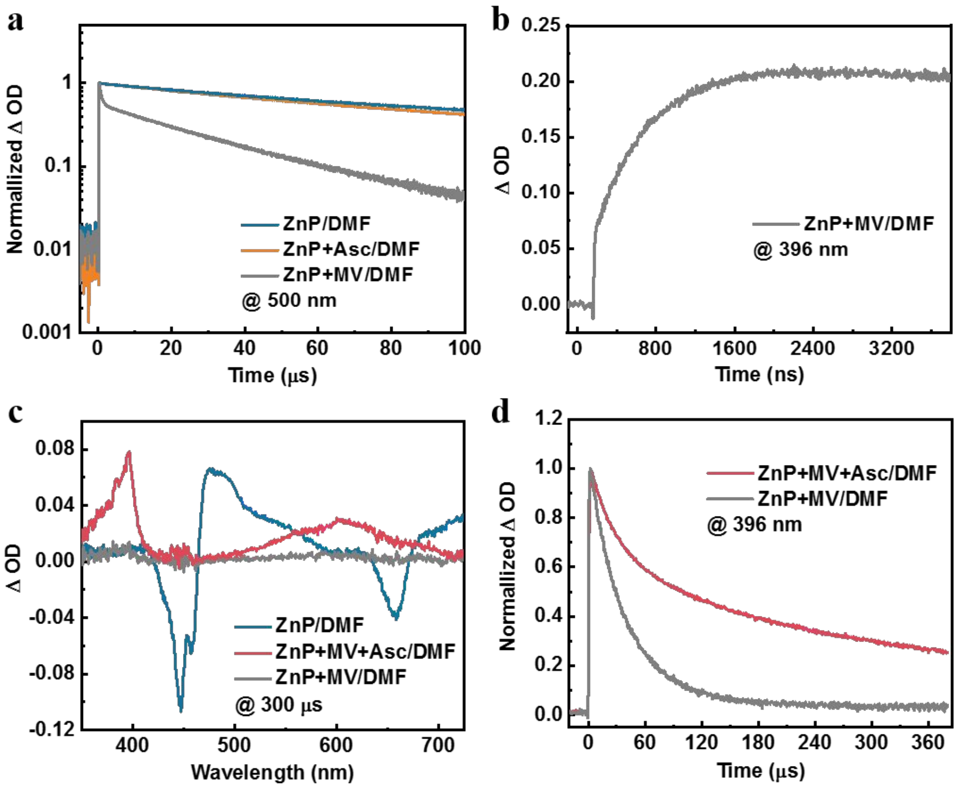

Figure S11. (a) Comparison of normalized TA kinetics probed at 500 nm of ZnP, ZnP with 0.2 M ascorbic acid, and ZnP with 1.6 mM methyl viologen in DMF; (b) TA kinetics probed at 396 nm of ZnP with 1.6 mM methyl viologen on the time scale of 4 μs; (c) Comparison of TA spectra monitored at a delay time of 300 μs of the ZnP, ZnP with 1.6 mM methyl viologen, ZnP with both 1.6 mM methyl viologen and 0.2 M ascorbic acid; (d) Normalized TA kinetics probed at 396 nm of ZnP with 1.6 mM methyl viologen, ZnP with both 1.6 mM methyl viologen and 0.2 M ascorbic acid.

Figure S7 showed TA spectra of the ZnP (ZnP/DMF), ZnP with 1.6 mM methyl viologen (ZnP+MV/DMF), ZnP with 0.2 M ascorbic acid (ZnP+Asc/DMF), and ZnP with both 1.6 mM methyl viologen and 0.2 M ascorbic acid (ZnP+MV+Asc/DMF) at several delay times after laser irradiation. The ns-TA spectra of ZnP revealed two GSBs minima at 450 and 650 nm, respectively. Photoinduced $T_1 \rightarrow T_n$ excited state absorption bands appear with maxima at 420, 500, and after 670 nm (Figure S7a). After adding 0.2 M ascorbic acid into the ZnP solution, it was found that the TA spectra (Figure S7b) and kinetics at 500 nm (Figure S8a) displayed negligible change. In contrast, as shown in Figure S7c, when 1.6 mM methyl viologen was added into the ZnP solution, the absorption band at around 500 nm decreased with the increasing delay time, and at the same time, three new absorption bands peaked at 396, 570, and 620 nm appeared. The 550 nm absorption band is assigned to ZnP^{+}, which is proved by spectroelectrochemistry data (Figure S9); 396 and 620 nm absorption bands are ascribed to the reduced viologen.[9, 10] A faster decay of ^{3}*ZnP at 500 nm after the addition of MV^{2+} (Figure S8a), with the increase of MV^{+} signal during the first 4000 ns at 396 nm (Figure S8b). Furthermore, ns-TA measurements were carried out in the presence of both 1.6 mM MV^{2+} and 0.2 M ascorbic acid, and the spectra are shown in Figure S7d. As shown in Figure S8c, the red line indicates the TAS of reduced MV. The addition of ascorbic acid leads to a prolongated lifetime of the MV^{+} from 40 μs to 205 μs (Figure S8d).

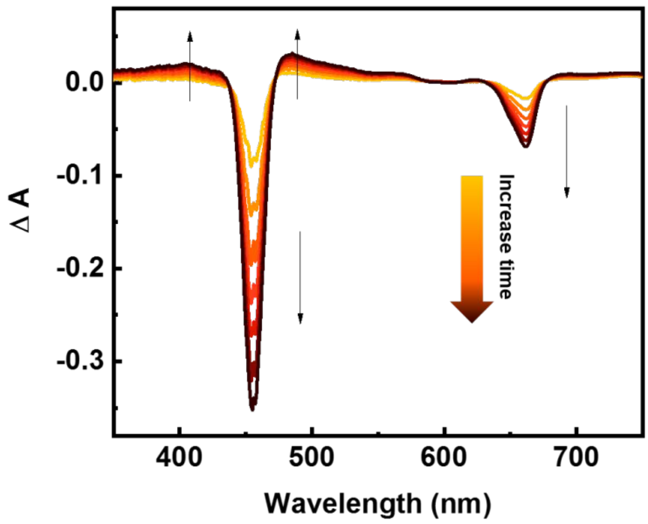

Figure S12. Spectroelectrochemistry of the ZnP measured in DMF, with a bias potential of 0.8 V (vs SHE).

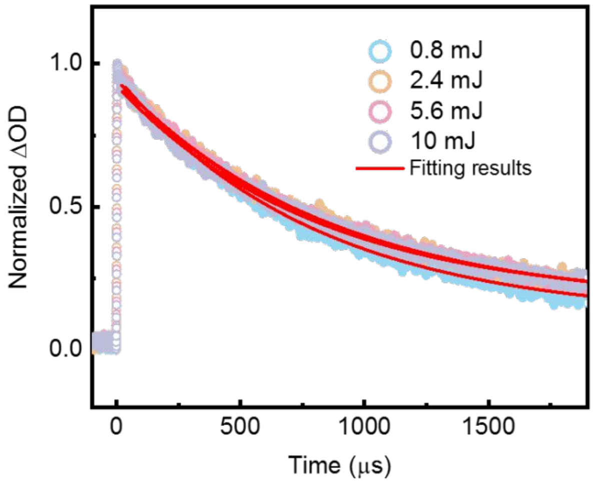

Figure S13. Normalized TA kinetics probed at 500 nm of ZnP in DMF, pump power (0.8 mJ/pulse, 2.4 mJ/pulse, 5.6 mJ/pulse, 10 mJ/pulse).

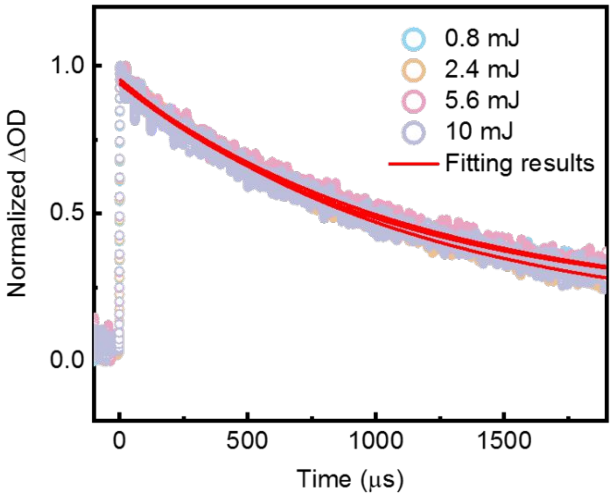

Figure S14. Normalized TA kinetics probed at 500 nm of ZnP-V in DMF, pump power (0.8 mJ/pulse, 2.4 mJ/pulse, 5.6 mJ/pulse, 10 mJ/pulse).

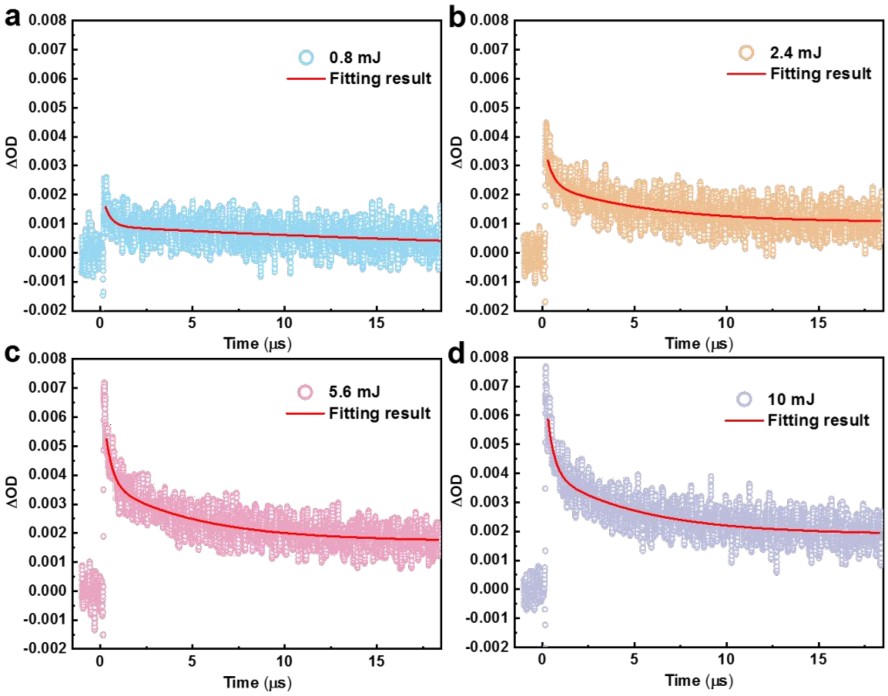

Figure S15. TA kinetics probed at 396 nm of ZnP-V nanoparticles in H_2O, pump power (0.8 mJ/pulse, 2.4 mJ/pulse, 5.6 mJ/pulse, 10 mJ/pulse).

Table S4. TA kinetics parameters probed at 396 nm of ZnP-V nanoparticles in H_2O with varied pump powers.

Power	A_1	A_2	t_1	weight	t_2	weight	Offset amplitude	Max amplitude	Offset/Max amplitude ratio
0.9 mJ	0.0011	0.0012	380 ns	47.8%	5.03 μs	52.2%	0.00047	0.00148	32%
2.4 mJ	0.0011	0.0013	380 ns	45.8%	5.48 μs	54.2%	0.00104	0.00301	35%
5.6 mJ	0.0011	0.0019	380 ns	36.7%	5.03 μs	63.3%	0.00164	0.00532	31%
10 mJ	0.0011	0.0019	380 ns	36.7%	5.08 μs	63.3%	0.00194	0.00585	33%

Fitting formula: $y = y_0 + A_1 e^{-(x-x_0)/t_1} + A_2 e^{-(x-x_0)/t_2}$

The data on the ns time scale shows only a small dependence on pump power: the initial signal only increases three-fold with a more than ten-fold increase in excitation power; at the same time, the lifetimes and relative amplitudes of the ns-μs components remain very similar. This suggests that sub-ns exciton annihilation may occur, but that the remaining exciton products at ns time scales and above are essentially independent of pump-power.

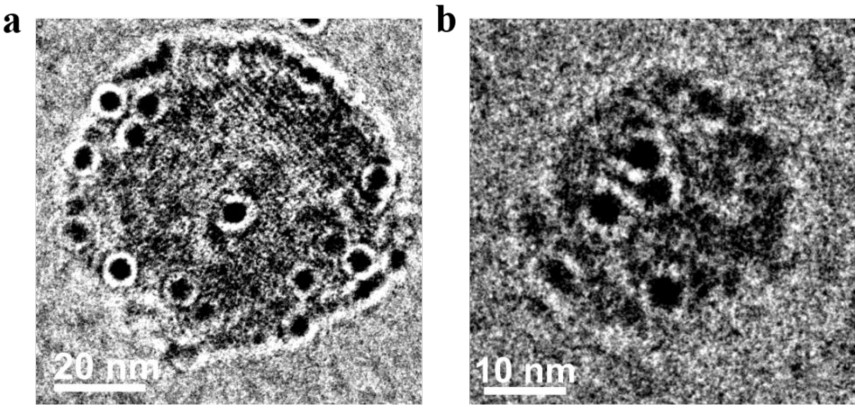

Figure S16. Cryo-EM micrographs of (a) ZnP nanoparticle and (b) ZnP-V nanoparticle with photo-deposited Pt.

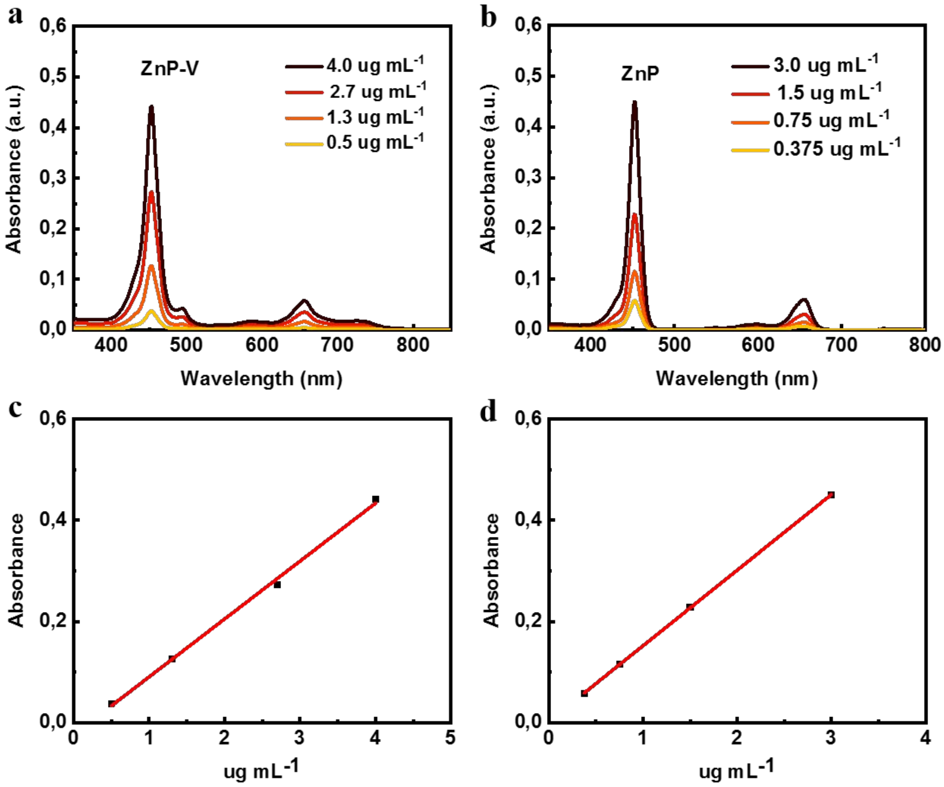

Figure S17. UV-vis absorption spectra of the (a) ZnP and (b) ZnP-V under different concentrations; a linear relationship of the absorbance at 450 nm of (c) ZnP and (d) ZnP-V.

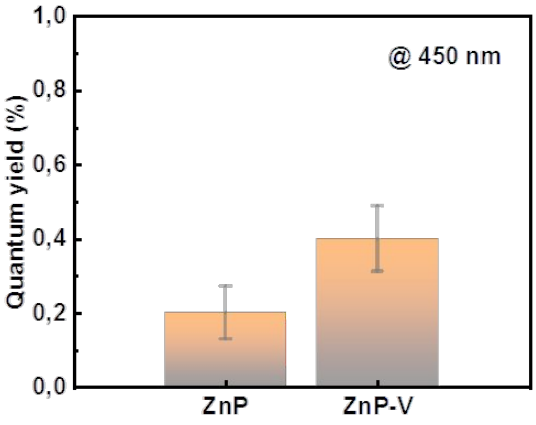

Figure S18. The external quantum yield of the H_2 production of the ZnP and ZnP-V nanoparticles was measured at 450 nm.

Table S5 Examples of the long CS states lifetime in Donor-Acceptor systems

System	Donor	Acceptor	CS state lifetime	Reference
Electrostatic assembly	Zinc porphyrin	Fullerene	1.1 μs	*J. Phys. Chem. B* 2003, 107, 13273[11]
Π-stacked self-assembly	Zinc porphyrin	Perylenediimide	169 ns	*Angew. Chem. Int. Ed.*, 2014, 53, 3457[12]
Tetrad	Tetra-alkylphenyldiamine	Naphthalene diimide	850 ns	*J. Am. Chem. Soc.*, 2016, 138, 11, 3752[13]
Ordered films	Diketopyrrolopyrrole	Perylenediimide	4 μs	*Chem. Sci.*, 2015 ,6, 402[14]
Heterojunction Nanoparticles	PM6 (polymer)	Y6	Second scale	*Nature Energy,* 2022, 7, 340[15]
Non-covalently linked assembly	Zinc porphyrin	Fullerene	220.9 ps	*Applied Catalysis B: Environmental,* 2023, 324, 122284[16]
Dyad Nanoparticles	Zinc porphyrin	viologen	4.3 ms	This work

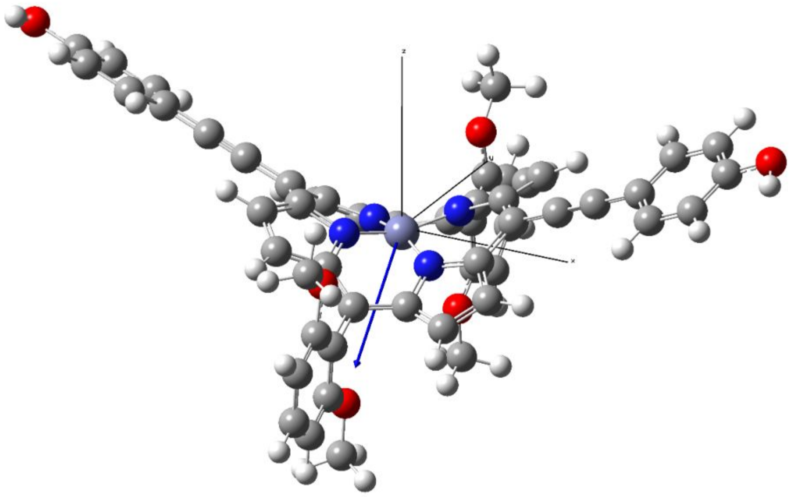

Figure S19. The dipole moment of the ground-state structure is 2.44 D and is oriented in the (y,z)-plane in the (-y,-z)-direction.

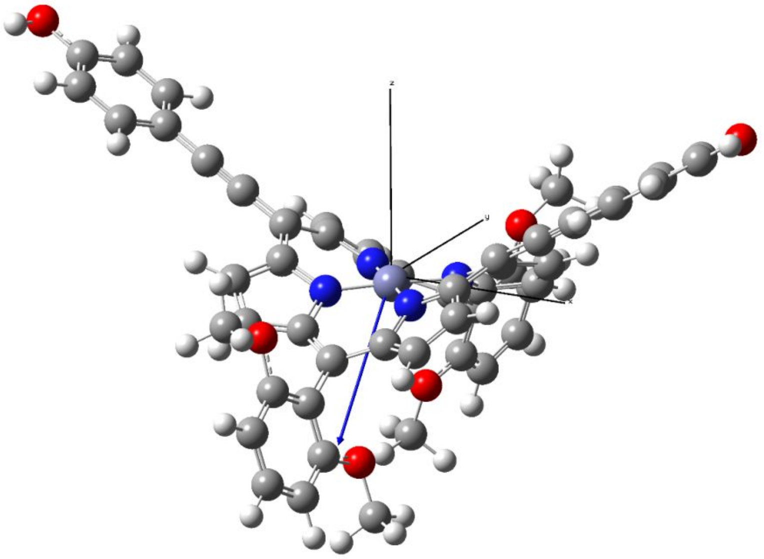

Figure S20. The 1st singlet excited state was geometrically optimized, showing a slight twist of the molecular groups in the y-direction. The dipole moment of the excited state structure has essentially the same direction as the ground state, but its dipole moment is significantly higher; 4.76 D.

The porphyrin model was studied using the hybrid density functional B3LYP implemented in Gaussian 16 (Rev. C.01)[17]. The molecular structure was geometrically optimized. Single-point time-dependent calculations (TD-DFT) were made on the geometrically optimized structures using the cam-B3LYP hybrid functional[18]. 6-31G(d,p) basis sets were used for all the light elements (H, C, N, O and S), and for Zn a small-core Stuttgart-Dresden-Cologne effective-core potential (MDF10) was employed in combination with a valence space of double-zeta quality[19].

Considering the high symmetry of the porphyrin systems in the study, we expect that any field-induced Stark effect would be small inside one molecule. In order to test this, TD-DFT computations were performed on a model porphyrin relevant to the studied systems (see

the calculations below). It should be noted that the model porphyrin with simplified structure was used in calculations as the long alkyl chains do not significantly affect the change in the porphyrin core's dipole moment. The observed transitions could be identified. The dipole moments of the optimized structures of the singlet ground state and the 1st excited singlet state are oriented similarly and the excitation results in a stronger dipole moment (from 2.44 D to 4.76 D). Considering that the charge hopping can occur in various directions within different molecule aggregation domains and that the molecular packing can differ across these domains, it is expected that the built electric field in the nanoparticle system will show different orientation and different strength. DFT calculations included two "ideal" electric field orientations (opposite and same direction to the change of dipole moment vector of the molecule at group and excited state) to verify the charge of the main transition. From the obtained calculations, we found that both applied electric fields shift the main transition by only 1-4 nm. These results verify the conclusion that the Stark effect is insignificant in these systems. However, we acknowledge that the situation within nanoparticles could be much more complicated than in the simplified model system used for these calculations. Therefore, although we did not see obvious Stark effect from the transient spectra, the charge hopping between molecules should not be excluded in the hypothesis, particularly because of the observed long-lived charge-separated state.

Reference

1. Yella, A.; Lee, H.-W.; Tsao, H. N.; Yi, C.; Chandiran, A. K.; Nazeeruddin, M. K.; Diau, E. W.-G.; Yeh, C.-Y.; Zakeeruddin, S. M.; Grätzel, M., Porphyrin-Sensitized Solar Cells with Cobalt (II/III), 2013;Based Redox Electrolyte Exceed 12 Percent Efficiency. *Science* **2011,** *334* (6056), 629-634.
2. Papadakis, R.; Deligkiozi, I.; Giorgi, M.; Faure, B.; Tsolomitis, A., Supramolecular complexes involving non-symmetric viologen cations and hexacyanoferrate(ii) anions. A spectroscopic, crystallographic and computational study. *RSC Advances* **2016,** *6* (1), 575-585.
3. Chang, J. P.; Fung, E. Y.; Curtis, J. C., Evidence for specific solvent-solute interactions as a major contributor to the Franck-Condon energy in intervalence-transfer absorptions of ruthenium ammine complexes. *Inorganic chemistry* **1986,** *25* (23), 4233-4241.
4. Wolf, M.; Lungerich, D.; Bauroth, S.; Popp, M.; Platzer, B.; Clark, T.; Anderson, H. L.; Jux, N.; Guldi, D. M., Panchromatic light funneling through the synergy in hexabenzocoronene-(metallo)porphyrin-fullerene assemblies to realize the separation of charges. *Chemical Science* **2020,** *11* (27), 7123-7132.
5. Fu, B.; Che, Y.; Yuan, X.; Sun, L.; Xu, H.; Zhao, J.; Liu, L., Photoinduced energy transfer in truxene-linked zinc porphyrin–fullerene-corrole tetrad and its application in triplet−triplet annihilation upconversion. *Dyes and Pigments* **2021,** *196*, 109754.
6. Togashi, D. M.; Costa, S. M. B., Excited state quenching kinetics of zinc meso-tetrakis (N-methylpyridinium-4-yl) porphyrin by methyl viologen in AOT reverse micelles. *Physical Chemistry Chemical Physics* **2002,** *4* (7), 1141-1150.
7. Smith, C. L.; Mears, L. L. E.; Greeves, B. J.; Draper, E. R.; Doutch, J.; Adams, D. J.; Cowan, A. J., Gelation enabled charge separation following visible light excitation using self-assembled perylene bisimides. *Physical Chemistry Chemical Physics* **2019,** *21* (48), 26466-26476.
8. Wu, Y.; Young, R. M.; Frasconi, M.; Schneebeli, S. T.; Spenst, P.; Gardner, D. M.; Brown, K. E.; Würthner, F.; Stoddart, J. F.; Wasielewski, M. R., Ultrafast Photoinduced Symmetry-Breaking Charge Separation and Electron Sharing in Perylenediimide Molecular Triangles. *Journal of the American Chemical Society* **2015,** *137* (41), 13236-13239.

9. Nie, Y.; Zhang, M.; Zhu, Y.; Jing, Y.; Shi, W.; Li, G.; Chen, H.; Jiang, Y.; Zhao, X.; Zhao, T.; Lu, G.; Li, S., Electrochromism of Viologen/Polymer Composite: From Gel to Insulating Bulk for High-Voltage Applications. *Materials* **2021,** *14* (19), 5901.
10. Kannappan, R.; Bucher, C.; Saint-Aman, E.; Moutet, J.-C.; Milet, A.; Oltean, M.; Métay, E.; Pellet-Rostaing, S.; Lemaire, M.; Chaix, C., Viologen-based redox-switchable anion-binding receptors. *New Journal of Chemistry* **2010,** *34* (7), 1373-1386.
11. Balbinot, D.; Atalick, S.; Guldi, D. M.; Hatzimarinaki, M.; Hirsch, A.; Jux, N., Electrostatic Assemblies of Fullerene−Porphyrin Hybrids: Toward Long-Lived Charge Separation. *The Journal of Physical Chemistry B* **2003,** *107* (48), 13273-13279.
12. Roznyatovskiy, V. V.; Carmieli, R.; Dyar, S. M.; Brown, K. E.; Wasielewski, M. R., Photodriven Charge Separation and Transport in Self-Assembled Zinc Tetrabenzotetraphenylporphyrin and Perylenediimide Charge Conduits. *Angewandte Chemie International Edition* **2014,** *53* (13), 3457-3461.
13. Favereau, L.; Makhal, A.; Pellegrin, Y.; Blart, E.; Petersson, J.; Göransson, E.; Hammarström, L.; Odobel, F., A Molecular Tetrad That Generates a High-Energy Charge-Separated State by Mimicking the Photosynthetic Z-Scheme. *Journal of the American Chemical Society* **2016,** *138* (11), 3752-3760.
14. Hartnett, P. E.; Dyar, S. M.; Margulies, E. A.; Shoer, L. E.; Cook, A. W.; Eaton, S. W.; Marks, T. J.; Wasielewski, M. R., Long-lived charge carrier generation in ordered films of a covalent perylenediimide–diketopyrrolopyrrole–perylenediimide molecule. *Chemical Science* **2015,** *6* (1), 402-411.
15. Kosco, J.; Gonzalez-Carrero, S.; Howells, C. T.; Fei, T.; Dong, Y.; Sougrat, R.; Harrison, G. T.; Firdaus, Y.; Sheelamanthula, R.; Purushothaman, B.; Moruzzi, F.; Xu, W.; Zhao, L.; Basu, A.; De Wolf, S.; Anthopoulos, T. D.; Durrant, J. R.; McCulloch, I., Generation of long-lived charges in organic semiconductor heterojunction nanoparticles for efficient photocatalytic hydrogen evolution. *Nature Energy* **2022,** *7* (4), 340-351.
16. Jing, J.; Li, J.; Su, Y.; Zhu, Y., Non-covalently linked donor-acceptor interaction enhancing photocatalytic hydrogen evolution from porphyrin assembly. *Applied Catalysis B: Environmental* **2023,** *324*, 122284.

17. M. J. Frisch, G. W. Trucks, H. B. Schlegel, G. E. Scuseria, M. A. Robb, J. R. Cheeseman, G. Scalmani, V. Barone, G. A. Petersson, H. Nakatsuji, X. Li, M. Caricato, A. V. Marenich, J. Bloino, B. G. Janesko, R. Gomperts, B. Mennucci, H. P. Hratchian, J. V. Ortiz, A. F. Izmaylov, J. L. Sonnenberg, D. Williams-Young, F. Ding, F. Lipparini, F. Egidi, J. Goings, B. Peng, A. Petrone, T. Henderson, D. Ranasinghe, V. G. Zakrzewski, J. Gao, N. Rega, G. Zheng, W. Liang, M. Hada, M. Ehara, K. Toyota, R. Fukuda, J. Hasegawa, M. Ishida, T. Nakajima, Y. Honda, O. Kitao, H. Nakai, T. Vreven, K. Throssell, J. A. Montgomery, Jr., J. E. Peralta, F. Ogliaro, M. J. Bearpark, J. J. Heyd, E. N. Brothers, K. N. Kudin, V. N. Staroverov, T. A. Keith, R. Kobayashi, J. Normand, K. Raghavachari, A. P. Rendell, J. C. Burant, S. S. Iyengar, J. Tomasi, M. Cossi, J. M. Millam, M. Klene, C. Adamo, R. Cammi, J. W. Ochterski, R. L. Martin, K. Morokuma, O. Farkas, J. B. Foresman, and D. J. Fox, Gaussian16 (Rev. B.01), Gaussian, Inc., Wallingford CT, 2016.
18. T. Yanai, D. Tew, N. Handy, *Chem. Phys. Lett.*, **393**, *2004*, 51-57.
19. D. Figgen, G. Rauhut, M. Dolg, H. Stoll, *Chem. Phys.*, **311**, *2005*, 227; K. A. Peterson, C. Puzzarini, *Theor. Chem. Acc.*, **114**, *2005*, 283.
